# Miracidia as main source for autofluorescence of *Schistosoma mansoni* eggs

**DOI:** 10.1590/0074-02760250215

**Published:** 2026-03-09

**Authors:** Danielle Segóvia Chrysóstomo de Almeida Pereira, Laila Oliveira Vaz Oliveira Oliveira, Felipe Tonon Firmino, Thomas Hanscheid, Rock Pulak, Malcolm Jones, Silvio Dolabella, Deborah Negrão-Corrêa, Carlos Graeff-Teixeira

**Affiliations:** 1 Universidade Federal do Espírito Santo, Núcleo de Doenças Infecciosas, Helmintologia, Vitória, ES, Brasil; 2 Universidade de Lisboa, Escola de Medicina, Lisboa, Portugal; 3 Union Biometric, Holliston, MA, United States of America; 4 University of Queensland, School of Veterinary Sciences, Gatton, Australia; 5 Universidade Federal de Sergipe, Centro de Ciências Biológicas e da Saúde, Entomologia e Parasitologia Tropical, Aracaju, SE, Brasil; 6 Universidade Federal de Minas Gerais, Instituto de Ciências Biológicas, Departamento de Parasitologia, Belo Horizonte, MG, Brasil

**Keywords:** schistosomiasis, autofluorescence, coproparasitology, Helmintex, egg

## Abstract

**BACKGROUND:**

Egg detection still has a role in schistosomiasis control, as a screening strategy or to provide a reference standard for the assessment of the accuracy of other diagnostic tools. The Helmintex method is highly sensitive but laborious, and several improvements of it, including automated egg detection, are currently under development.

**OBJECTIVE:**

We conducted a preliminary evaluation of *Schistosoma mansoni* eggs’ autofluorescence as a distinctive marker amid very complex fecal sediments.

**METHODS:**

Eggs from mouse livers and human feces were examined under a fluorescence microscope.

**FINDINGS:**

More intense green fluorescence (greater for miracidia than for eggshell) was consistently detected using a B-2A filter (FITC, 420-495 nm).

**MAIN CONCLUSIONS:**

These findings may help to improve diagnostic methods, especially with automated egg detection systems. Besides access to safe water and adequate sanitation, as well as health education and the treatment of infected individuals, laboratory diagnosis is a key measure that can help eliminate schistosomiasis as a public health problem.

Although the sensitivity limitations of egg detection–based methods for the diagnosis of parasitic infections are well known, the Kato-Katz fecal thick smear is still used widely in many endemic areas for the diagnosis of schistosomiasis. Due to eggs’ peculiar size and morphology,^1^ their identification in fecal samples enables highly specific classification, what is valuable for the establishment of reliable reference data for the assessment of other diagnostic tools’ accuracy. The Helmintex (HTX) method involves the use of magnetism to isolate *Schistosoma mansoni* eggs from feces; it has 100% sensitivity for egg burdens > 1.3 eggs per gram.^2^ Despite its accuracy, this method is labor intensive, and several improvements are currently underway. One promising possibility is a detection step using a flow system coupled with morphometric and fluorescent detectors. We thus investigated the autofluorescence of *S. mansoni* eggs to aid their identification in ongoing flow system detector development.

## MATERIALS AND METHODS

*Schistosoma mansoni* eggs were obtained from (i) experimentally infected BALB/c mouse livers (ML) (CEUA-UFMG ethical approval 41/2024) and (ii) sediment pools from naturally infected human feces (HF) from a biorepository under proper ethical clearance (CEP-CCS-UFES CAAE 71027323.7.0000.5060). Eggs isolated from ML after seven-eight weeks post infection were kept in 10% formalin solution,^3^ and those isolated from HF were fixed in 50% alcohol and stored at 10ºC.

**Fig. 1:**
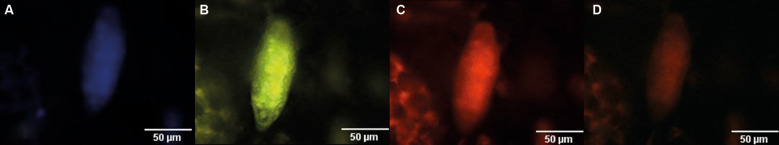
autofluorescence of *Schistosoma mansoni* eggs from human fecal samples under microscope filters: (A) UV-2E (330-380 nm), Dichroic Mirror (DM) 400 nm, emission: > 420 longpass (LP), Blue; (B) B-2A (FITC, 420-495 nm), DM 505 nm, emission: > 515 LP, Green/Yellow; (C) G-2A(TRITC, 510-560 nm), 575 nm, emission: > 590 LP, Orange/Red; and (D) Y-2E/C (540-580 nm), 595 nm, emission: > 630 LP, Red.

Eggs from MLs (n = 20) were inoculated into 30 µL uninfected HF sediment produced by the HTX method.^4^ Briefly, the HTX steps were sequential passage through 500-, 150- and 45-µm sieves and centrifugation with ethyl-acetate in water. The sediment with eggs was spread onto microscope slides and left to dry at room temperature. The same procedure was performed with three sets containing 12 to 14 HF eggs. The slides were examined under a fluorescence microscope (Eclipse 80i, LED light source; Nikon Corporation, Tokyo, Japan) with the filter sets indicated in Fig. 1.

## RESULTS

**Fig. 2:**
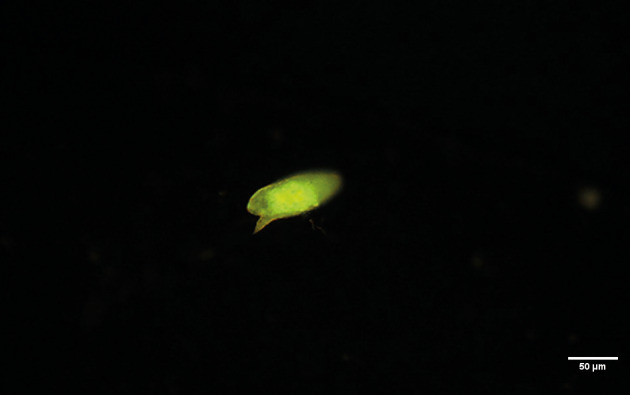
*Schistosoma mansoni* egg obtained from the liver of an experimentally infected mouse under B-2A (FITC) filter (420-495 nm, blue; emission: > 515 nm longpass) showing miracidium as the source of a more intense autofluorescence.

All eggs from MLs and HF exhibited visible autofluorescence under the B-2A filter, with the miracidium having the most intense signal and the eggshell having a weaker signal (Fig. 2). Less-intense fluorescence was observed with the other filters (Fig. 1). These findings suggest that *S. mansoni* egg autofluorescence originates primarily from functional molecules in the miracidium, rather than from structural components such as the eggshell.

## DISCUSSION

Autofluorescence is a natural optical property observed in the tissues and cellular organelles of organisms in various taxonomic groups.^5^ ^6^ The autofluorescence of *S. mansoni* eggs in murine and human tissues (e.g., liver and gut) and eggshells released by or located in the reproductive organs of in vitro-cultivated worms have been reported^7^ ^,^ ^8^ ^,^ ^9^ ^,^ ^10^ ^,^ ^11^ (Table). In all these reports, the eggshell is identified as the source of fluorescence, although compelling evidence is lacking and some images do not clearly show fluorescent structures. In addition, these studies did not focus on the examination of parasite structure autofluorescence, which was reported as an undesired side effect in some cases.^12^ ^13^

Sites of structural protein cross-linkage have been considered to be the main sites of autofluorescence in trematode eggshells.^14^ We observed prominent autofluorescence signals from *S. mansoni* egg miracidia, suggesting that metabolically active or intact miracidia are the main contributors (Fig. 2). Contrary to our findings, most reports describe autofluorescence only in *S. mansoni* eggshells, which may be explained by the performance of procedures with whole worms or tissues that remove miracidia contents or prevent their autofluorescence. The similarity of our findings for formaldehyde-fixed eggs from MLs and alcohol-fixed eggs from HF does not support the potential for the degradation or removal of the molecular source of autofluorescence by fixatives or reduced pH.^15^

**TABLE:** Reports from the literature describing autofluorescent *Schistosoma* spp. eggs

Reference	Egg sources			Main fluorescent structure
	Mice tissue	*In vitro* culture	Human tissue	
Domingo 1968	rectum / liver		rectum	eggshell
Edwards 2015		inside the worm		eggshell / miracidia (?)
Wang 2018		released by worms		eggshell
Knhur 2018	gut / liver			eggshell
Peterkova 2024	liver			eggshell

A distinctive noise-to-signal ratio is important when using autofluorescence to detect infectious agents, as the present results demonstrate.^13^ The distinct fluorescence signature of the miracidium, especially under the B-2A filter, is a reliable marker that stands out among fecal debris. Such markers are essential for the development of automated detection systems, but they may also improve detection by conventional microscopy. Autofluorescence is not commonly used to detect parasites amid very diverse fecal debris. Sakurai and collaborators^16^ submitted a patent for a procedure involving the use of autofluorescence to detect *Schistosoma japonicum* eggs.

In conclusion, we report that *S. mansoni* eggs autofluorescence in fecal debris, enabling their relatively clear distinction from the background. We provide compelling evidence that this autofluorescence originates mainly from miracidia, and not eggshells, contrasting with most reports in the literature. These findings support the hypothesis that functional, rather than structural, molecules are the primary source of autofluorescence in mature *S. mansoni* eggs.

## Data Availability

The contents underlying the research text are included in the manuscript.
